# Supplementary material for: Computational analysis of the LRRK2 interactome
Source: PeerJ. 2015 Feb 19;3:e778. doi: 10.7717/peerj.778 (PMC4338795; doi:10.7717/peerj.778)
Supplement: Table S1 — It contains details regarding the merging of 6 problematic records duplicated differently between the BioGRID and IntAct repositories. Annotations in white has been kept in the final record moved into the merged dataset, annotations in red has been removed. (A).Rationale: (i) Protein Kinase Assay = Biochemical Activity (one of the redundant entries has been removed); (ii) Affinity Capture-Western (BioGRID) is split in Pull Down and Anti-tag Coimmunoprecipitation in IntAct, following the rule of keeping the record with maximum number of annotations, the record in IntAct has been preferred over the one in BioGRID. (B). Rationale: (i) Protein Kinase Assay = Biochemical Activity (one of the redundant entries has been removed). (C). Rationale: (i) Affinity Capture-Western = Affinity Chromatography Technology (one of the redundant entries has been removed). (D).Rationale : (i) Affinity Capture-Western = Anti-tag Coimmunoprecipitation (one of the redundant entries has been removed). (E). Rationale: (i) Affinity Capture-Western/MS = Pull Down (one of the redundant entries has been removed). (F). Rationale: Merged. [file peerj-03-778-s001.docx]

| Database | Protein | Author | PMD id | Method |
| --- | --- | --- | --- | --- |
| BioGRID | **TUBA1A** | Kawakami F (2012) | 22303461 | Affinity Capture-Western |
| BioGRID | **MAPT** | Kawakami F (2012) | 22303461 | Affinity Capture-Western |
| BioGRID | **TUBA1A** | Kawakami F (2012) | 22303461 | Biochemical Activity |
| BioGRID | **MAPT** | Kawakami F (2012) | 22303461 | Biochemical Activity |
| IntAct | **MAPT** | Kawakami F (2012) | 22303461 | Protein Kinase Assay |
| IntAct | **MAPT** | Kawakami F (2012) | 22303461 | Pull Down |
| IntAct | **MAPT** | Kawakami F (2012) | 22303461 | Anti-tag Coimmunoprecipitation |

**A**

**B**

| Database | Protein | Author | PMD id | Method |
| --- | --- | --- | --- | --- |
| BioGRID | **AKT1** | Ohta E (2011) | 21658387 | Biochemical Activity |
| BioGRID | **AKT1** | Ohta E (2011) | 21658387 | Reconstituted Complex |
| IntAct | **AKT1** | Ohta E (2011) | 21658387 | Protein Kinase Assay |
| IntAct | **AKT1** | Ohta E (2011) | 21658387 | Pull Down |

**C**

| Database | Protein | Author | PMD id | Method |
| --- | --- | --- | --- | --- |
| BioGRID | **HSP90AA1** | Gloeckner CJ (2006) | 16321986 | Affinity Capture-Western |
| BioGRID | **CDC37** | Gloeckner CJ (2006) | 16321986 | Affinity Capture-Western |
| IntAct | **CDC37** | Gloeckner CJ (2006) | 16321986 | Affinity Chromatography Technology |
| IntAct | **CDC37** | Gloeckner CJ (2006) | 16321986 | Tandem Affinity Purification |

**D**

| Database | Protein | Author | PMD id | Method |
| --- | --- | --- | --- | --- |
| BioGRID | **MAP2K6** | Hsu CH (2010) | 20067578 | Affinity Capture-Western |
| BioGRID | **MAP2K7** | Hsu CH (2010) | 20067578 | Affinity Capture-Western |
| BioGRID | **MAP2K7** | Hsu CH (2010) | 20067578 | Biochemical Activity |
| BioGRID | **MAP2K6** | Hsu CH (2010) | 20067578 | Biochemical Activity |
| BioGRID | **MAP2K3** | Hsu CH (2010) | 20067578 | Biochemical Activity |
| IntAct | **MAP2K6** | Hsu CH (2010) | 20067578 | Anti-tag Coimmunoprecipitation |
| IntAct | **MAP2K3** | Hsu CH (2010) | 20067578 | Anti-tag Coimmunoprecipitation |
| IntAct | **MAP2K7** | Hsu CH (2010) | 20067578 | Anti-tag Coimmunoprecipitation |
| IntAct | **MAP2K6** | Hsu CH (2010) | 20067578 | Fluorescence Microscopy |

**E**

| Database | Protein | Author | PMD id | Method |
| --- | --- | --- | --- | --- |
| BioGRID | **YWHAZ** | Nichols RJ (2010) | 20642453 | Affinity Capture-Western |
| BioGRID | **YWHAE** | Nichols RJ (2010) | 20642453 | Affinity Capture-Western |
| BioGRID | **YWHAH** | Nichols RJ (2010) | 20642453 | Affinity Capture-Western |
| BioGRID | **YWHAB** | Nichols RJ (2010) | 20642453 | Affinity Capture-Western |
| BioGRID | **YWHAG** | Nichols RJ (2010) | 20642453 | Affinity Capture-Western |
| BioGRID | **YWHAZ** | Nichols RJ (2010) | 20642453 | Affinity Capture-MS |
| BioGRID | **YWHAQ** | Nichols RJ (2010) | 20642453 | Affinity Capture-MS |
| BioGRID | **YWHAE** | Nichols RJ (2010) | 20642453 | Affinity Capture-MS |
| BioGRID | **YWHAB** | Nichols RJ (2010) | 20642453 | Affinity Capture-MS |
| BioGRID | **YWHAH** | Nichols RJ (2010) | 20642453 | Affinity Capture-MS |
| BioGRID | **CDC37** | Nichols RJ (2010) | 20642453 | Affinity Capture-MS |
| BioGRID | **HSP90AA1** | Nichols RJ (2010) | 20642453 | Affinity Capture-MS |
| BioGRID | **HSP90AB1** | Nichols RJ (2010) | 20642453 | Affinity Capture-MS |
| BioGRID | **YWHAG** | Nichols RJ (2010) | 20642453 | Affinity Capture-MS |
| BioGRID | **YWHAQ** | Nichols RJ (2010) | 20642453 | Affinity Capture-Western |
| IntAct | **YWHAQ** | Nichols RJ (2010) | 20642453 | Pull Down |
| IntAct | **YWHAB** | Nichols RJ (2010) | 20642453 | Pull Down |
| IntAct | **YWHAH** | Nichols RJ (2010) | 20642453 | Pull Down |
| IntAct | **YWHAZ** | Nichols RJ (2010) | 20642453 | Pull Down |
| IntAct | **YWHAE** | Nichols RJ (2010) | 20642453 | Pull Down |
| IntAct | **YWHAG** | Nichols RJ (2010) | 20642453 | Pull Down |
| IntAct | **MSN** | Nichols RJ (2010) | 20642453 | Protein Kinase Assay |

| Database | Protein | Author | PMD id | Method |
| --- | --- | --- | --- | --- |
| BioGRID | **EEF1A1** | Gillardon F (2009) | 19559761 | Affinity Capture-Western |
| IntAct | **EEF1A2** | Gillardon F (2009) | 19559761 | Protein Kinase Assay |

**F**
