# Supplementary material for: Computational analysis of the LRRK2 interactome
Source: PeerJ. 2015 Feb 19;3:e778. doi: 10.7717/peerj.778 (PMC4338795; doi:10.7717/peerj.778)
Supplement: Table S2 — It contains details regarding the 207 LRRK2 interactors that were annotated 1 time only. [file peerj-03-778-s002.docx]

| ABL1 | CUEDC1 | HMMR | PPP1R8 | RPS3 | TUBB2B |
| --- | --- | --- | --- | --- | --- |
| ABL2 | CWC15 | HOGA1 | PPP2R1A | RPS8 | TUBB3 |
| ACTA2 | CXCL11 | HSPA4 | PRKACA | SCFD1 | TUBB4B |
| ACTBL2 | DAPK1 | HSPA9 | PRKCZ | SFN | TUBB6 |
| ACTG1 | DAPP1 | HSPD1 | PSMD11 | SH3GL1 | UFD1L |
| ACTG2 | DBF4B | IQGAP1 | PSMG1 | SH3GL2 | VASH2 |
| ACTR2 | DBN1 | IRAK1BP1 | PTPN23 | SH3GL3 | WIBG |
| AKAP8 | DFFB | IRS1 | RAB11FIP2 | SLC25A4 | WIPF1 |
| AKT2 | DNAJA1 | L3MBTL3 | RAI14 | SLC25A5 | XIRP2 |
| ANKS4B | DPYSL3 | LDHB | RALYL | SLC25A6 | ZFAND5 |
| ARPC1B | DUX3 | LGALS8 | RBBP8 | SMEK1 | ZSCAN26 |
| ARPC2 | DYNC1H1 | LIMA1 | RGS1 | SNX9 | ZZZ3 |
| ARPC4 | EEF1A1 | LIMS2 | RHNO1 | SP100 |  |
| ARPC5 | EEF1A2 | MAP2K4 | RHOA | SPATA24 |  |
| ATP5L | EFHD2 | MAPK8IP2 | RIPK1 | STAC |  |
| AURKC | ENKUR | MDN1 | RIPK2 | STIP1 |  |
| BAG1 | EOGT | MKNK2 | RNF219 | STK24 |  |
| BRP44 | EPS8L2 | MLLT3 | RPAP3 | STK25 |  |
| C16orf80 | ERG | MPRIP | RPL10a | STK3 |  |
| C5orf45 | ETV5 | MRGBP | RPL10a | STK33 |  |
| C8orf59 | FAAP24 | MTA1 | RPL13 | STK40 |  |
| CALM1 | FADD | MYL12B | RPL14 | STRBP |  |
| CALM2 | FAM107A | MYL6 | RPL17 | SUDS3 |  |
| CALM3 | FAM27E3 | MYO1B | RPL21 | TAF7L |  |
| CAMK1D | FAM47B | MYO1C | RPL30 | TAOK3 |  |
| CAPZA1 | FIS1 | MYO1F | RPL34 | TCF25 |  |
| CAPZA2 | FLJ45872 | NDUFAF7 | RPL36a | TEX33 |  |
| CAPZB | FZD5 | NFATC2 | RPL39 | TMOD3 |  |
| CASP8 | GEMIN8 | NKRF | RPS11 | TNPO1 |  |
| CCDC43 | GIMAP8 | NRON | RPS13 | TOR1AIP2 |  |
| CCL21 | GLTPD1 | NSL1 | RPS15 | TP53TG3 |  |
| CD2BP2 | GNA12 | NUP133 | RPS15 | TPM2 |  |
| CDC25A | GNA13 | PADI4 | RPS15 | TPM3 |  |
| CDC42EP3 | GNAI2 | PARK2 | RPS16 | TRADD |  |
| CEP72 | GPBP1L1 | PDCD4 | RPS18 | TRAF2 |  |
| CHERP | H3F3A | PLEC | RPS2 | TRIB2 |  |
| CLTC | HIST1H3A | PLK1 | RPS20 | TTK |  |
| CMAS | HIST2H3B | POLE | RPS23 | TUBB1 |  |
| CSE1L | HMGN2 | PPP1R12A | RPS27 | TUBB2A |  |
