# Supplementary material for: Computational analysis of the LRRK2 interactome
Source: PeerJ. 2015 Feb 19;3:e778. doi: 10.7717/peerj.778 (PMC4338795; doi:10.7717/peerj.778)
Supplement: Table S3 — It contains details regarding grouping of the terms enriched in WebGestalt. [file peerj-03-778-s003.docx]

| transport /localization | |
| --- | --- |
| cellular localization | GO:0051641 |
| intracellular transport | GO:0046907 |
| establishment of localization in cell | GO:0051649 |
| cytoskeleton-dependent intracellular transport | GO:0030705 |
| cell organization | |
| cellular component organization at cellular level | GO:0071842 |
| cellular component organization | GO:0016043 |
| cellular component organization or biogenesis at cellular level | GO:0071841 |
| cellular component organization or biogenesis | GO:0071840 |
| regulation of kinase activity | |
| regulation of kinase activity | GO:0043549 |
| regulation of transferase activity | GO:0051338 |
| cytosol | |
| cytosol | GO:0005829 |
| cytoplasmic part | GO:0044444 |
| cytoplasm | GO:0005737 |
| vesicles | |
| cytoplasmic vesicle | GO:0031410 |
| vesicle | GO:0031982 |
| cytoplasmic membrane-bounded vesicle | GO:0016023 |
| membrane-bounded vesicle | GO:0031988 |
| cytoskeleton | |
| cytoskeleton | GO:0005856 |
| cell projections | |
| cell projection | GO:0042995 |
| neuron projection | GO:0043005 |
