# Supplementary material for: Computational analysis of the LRRK2 interactome
Source: PeerJ. 2015 Feb 19;3:e778. doi: 10.7717/peerj.778 (PMC4338795; doi:10.7717/peerj.778)
Supplement: Table S4 — It contains details regarding grouping of the terms enriched in Panther. BF, background frequency; SF, sample frequency. [file peerj-03-778-s004.docx]

| **Group** | **Term** | **Adjusted**  **p-value** | **BF** | **SF** |
| --- | --- | --- | --- | --- |
| **catabolism** | catabolic process (GO:0009056) | 8.79E-07 | 2075 | 23 |
| **catabolism** | organic substance catabolic process (GO:1901575) | 7.30E-07 | 1870 | 22 |
| **catabolism** | organic cyclic compound catabolic process (GO:1901361) | 1.76E-08 | 950 | 18 |
| **catabolism** | aromatic compound catabolic process (GO:0019439) | 8.70E-08 | 914 | 17 |
| **catabolism** | cellular nitrogen compound catabolic process (GO:0044270) | 8.55E-08 | 913 | 17 |
| **catabolism** | heterocycle catabolic process (GO:0046700) | 8.70E-08 | 914 | 17 |
| **catabolism** | nucleobase-containing compound catabolic process (GO:0034655) | 3.97E-08 | 868 | 17 |
| **catabolism** | carbohydrate derivative catabolic process (GO:1901136) | 8.65E-08 | 667 | 15 |
| **catabolism** | glycosyl compound catabolic process (GO:1901658) | 5.95E-09 | 549 | 15 |
| **catabolism** | nucleoside catabolic process (GO:0009164) | 5.11E-09 | 543 | 15 |
| **catabolism** | nucleoside phosphate catabolic process (GO:1901292) | 1.00E-08 | 570 | 15 |
| **catabolism** | nucleoside triphosphate catabolic process (GO:0009143) | 2.38E-09 | 514 | 15 |
| **catabolism** | nucleotide catabolic process (GO:0009166) | 8.64E-09 | 564 | 15 |
| **catabolism** | organophosphate catabolic process (GO:0046434) | 2.38E-08 | 607 | 15 |
| **catabolism** | purine nucleoside catabolic process (GO:0006152) | 2.80E-09 | 520 | 15 |
| **catabolism** | purine nucleoside triphosphate catabolic process (GO:0009146) | 2.19E-09 | 511 | 15 |
| **catabolism** | purine nucleotide catabolic process (GO:0006195) | 5.80E-09 | 548 | 15 |
| **catabolism** | purine ribonucleoside catabolic process (GO:0046130) | 2.80E-09 | 520 | 15 |
| **catabolism** | purine ribonucleoside triphosphate catabolic process (GO:0009207) | 2.02E-09 | 508 | 15 |
| **catabolism** | purine ribonucleotide catabolic process (GO:0009154) | 4.05E-09 | 534 | 15 |
| **catabolism** | purine-containing compound catabolic process (GO:0072523) | 6.58E-09 | 553 | 15 |
| **catabolism** | ribonucleoside catabolic process (GO:0042454) | 3.65E-09 | 530 | 15 |
| **catabolism** | ribonucleoside triphosphate catabolic process (GO:0009203) | 2.02E-09 | 508 | 15 |
| **catabolism** | ribonucleotide catabolic process (GO:0009261) | 4.15E-09 | 535 | 15 |
| **catabolism** | GTP catabolic process (GO:0006184) | 3.89E-10 | 226 | 12 |
| **catabolism** | guanosine-containing compound catabolic process (GO:1901069) | 4.52E-10 | 229 | 12 |
| **cell death** | cell death (GO:0008219) | 3.41E-11 | 1249 | 23 |
| **cell death** | death (GO:0016265) | 3.64E-11 | 1253 | 23 |
| **cell death** | apoptotic process (GO:0006915) | 6.45E-11 | 1021 | 21 |
| **cell death** | programmed cell death (GO:0012501) | 9.16E-11 | 1040 | 21 |
| **cell death** | regulation of apoptotic process (GO:0042981) | 2.89E-09 | 1249 | 21 |
| **cell death** | regulation of cell death (GO:0010941) | 7.32E-09 | 1313 | 21 |
| **cell death** | regulation of programmed cell death (GO:0043067) | 3.51E-09 | 1262 | 21 |
| **cell death** | apoptotic signaling pathway (GO:0097190) | 1.85E-09 | 332 | 13 |
| **cell organization** | cellular component organization (GO:0016043) | 4.63E-15 | 4259 | 43 |
| **cell organization** | cellular component organization or biogenesis (GO:0071840) | 1.20E-14 | 4366 | 43 |
| **cell organization** | organelle organization (GO:0006996) | 2.37E-07 | 2316 | 25 |
| **cell organization** | regulation of cellular component organization (GO:0051128) | 4.32E-11 | 1555 | 25 |
| **cell organization** | positive regulation of cellular component organization (GO:0051130) | 3.81E-11 | 654 | 18 |
| **cell organization** | cellular component morphogenesis (GO:0032989) | 4.98E-08 | 881 | 17 |
| **cell organization** | regulation of organelle organization (GO:0033043) | 1.03E-08 | 679 | 16 |
| **cell organization** | cell part morphogenesis (GO:0032990) | 8.82E-08 | 668 | 15 |
| **cell organization** | positive regulation of organelle organization (GO:0010638) | 3.64E-10 | 291 | 13 |
| **development** | developmental process (GO:0032502) | 2.09E-09 | 4643 | 38 |
| **development** | single-organism developmental process (GO:0044767) | 8.71E-09 | 4596 | 37 |
| **development** | anatomical structure development (GO:0048856) | 1.13E-09 | 4056 | 36 |
| **development** | system development (GO:0048731) | 1.10E-07 | 3498 | 31 |
| **development** | cellular developmental process (GO:0048869) | 2.11E-07 | 2917 | 28 |
| **development** | nervous system development (GO:0007399) | 3.43E-10 | 1873 | 26 |
| **development** | generation of neurons (GO:0048699) | 1.44E-09 | 1203 | 21 |
| **development** | neurogenesis (GO:0022008) | 4.31E-09 | 1276 | 21 |
| **development** | cell development (GO:0048468) | 9.55E-07 | 1380 | 19 |
| **development** | cell projection organization (GO:0030030) | 4.48E-09 | 873 | 18 |
| **development** | cell projection morphogenesis (GO:0048858) | 6.61E-07 | 653 | 14 |
| **development** | regulation of nervous system development (GO:0051960) | 4.76E-07 | 525 | 13 |
| **development** | regulation of cell projection organization (GO:0031344) | 4.65E-07 | 331 | 11 |
| **development** | substantia nigra development (GO:0021762) | 5.41E-07 | 42 | 6 |
| **development** | subthalamus development (GO:0021539) | 7.12E-07 | 44 | 6 |
| **general terms** | single-organism process (GO:0044699) | 7.69E-13 | 11989 | 61 |
| **general terms** | cellular process (GO:0009987) | 5.43E-09 | 13224 | 60 |
| **general terms** | single-organism cellular process (GO:0044763) | 3.42E-13 | 10603 | 59 |
| **general terms** | biological regulation (GO:0065007) | 1.52E-07 | 10036 | 52 |
| **general terms** | regulation of biological process (GO:0050789) | 2.00E-08 | 9585 | 52 |
| **general terms** | regulation of cellular process (GO:0050794) | 7.90E-08 | 9099 | 50 |
| **general terms** | positive regulation of biological process (GO:0048518) | 2.15E-11 | 4029 | 38 |
| **general terms** | positive regulation of cellular process (GO:0048522) | 1.43E-09 | 3615 | 34 |
| **general terms** | negative regulation of biological process (GO:0048519) | 2.41E-08 | 3522 | 32 |
| **general terms** | negative regulation of cellular process (GO:0048523) | 4.16E-07 | 3226 | 29 |
| **general terms** | regulation of molecular function (GO:0065009) | 1.65E-08 | 2417 | 27 |
| **immune response** | immune system process (GO:0002376) | 1.23E-07 | 1874 | 23 |
| **immune response** | immune response (GO:0006955) | 5.66E-09 | 1151 | 20 |
| **immune response** | defense response (GO:0006952) | 2.01E-07 | 1255 | 19 |
| **immune response** | innate immune response (GO:0045087) | 6.24E-10 | 774 | 18 |
| **immune response** | regulation of immune system process (GO:0002682) | 1.56E-07 | 1089 | 18 |
| **immune response** | regulation of immune response (GO:0050776) | 2.02E-08 | 711 | 16 |
| **immune response** | immune response-regulating signaling pathway (GO:0002764) | 7.77E-11 | 403 | 15 |
| **immune response** | immune response-regulating cell surface receptor signaling pathway (GO:0002768) | 5.76E-10 | 302 | 13 |
| **membrane processes** | membrane organization (GO:0061024) | 3.67E-14 | 695 | 21 |
| **metabolism** | phosphate-containing compound metabolic process (GO:0006796) | 4.21E-11 | 2218 | 29 |
| **metabolism** | phosphorus metabolic process (GO:0006793) | 6.78E-11 | 2260 | 29 |
| **metabolism** | regulation of protein metabolic process (GO:0051246) | 4.70E-10 | 1899 | 26 |
| **metabolism** | regulation of cellular protein metabolic process (GO:0032268) | 1.79E-11 | 1495 | 25 |
| **metabolism** | regulation of phosphate metabolic process (GO:0019220) | 4.70E-09 | 1754 | 24 |
| **metabolism** | regulation of phosphorus metabolic process (GO:0051174) | 5.48E-09 | 1767 | 24 |
| **metabolism** | glycosyl compound metabolic process (GO:1901657) | 5.73E-08 | 764 | 16 |
| **metabolism** | nucleoside metabolic process (GO:0009116) | 4.14E-08 | 747 | 16 |
| **metabolism** | nucleoside phosphate metabolic process (GO:0006753) | 6.87E-07 | 909 | 16 |
| **metabolism** | nucleoside triphosphate metabolic process (GO:0009141) | 4.97E-09 | 646 | 16 |
| **metabolism** | nucleotide metabolic process (GO:0009117) | 6.35E-07 | 904 | 16 |
| **metabolism** | positive regulation of cellular protein metabolic process (GO:0032270) | 8.78E-07 | 925 | 16 |
| **metabolism** | purine nucleoside metabolic process (GO:0042278) | 1.71E-08 | 703 | 16 |
| **metabolism** | purine nucleoside triphosphate metabolic process (GO:0009144) | 3.28E-09 | 628 | 16 |
| **metabolism** | purine nucleotide metabolic process (GO:0006163) | 4.65E-08 | 753 | 16 |
| **metabolism** | purine ribonucleoside metabolic process (GO:0046128) | 1.61E-08 | 700 | 16 |
| **metabolism** | purine ribonucleoside triphosphate metabolic process (GO:0009205) | 2.85E-09 | 622 | 16 |
| **metabolism** | purine ribonucleotide metabolic process (GO:0009150) | 3.08E-08 | 732 | 16 |
| **metabolism** | purine-containing compound metabolic process (GO:0072521) | 9.98E-08 | 794 | 16 |
| **metabolism** | ribonucleoside metabolic process (GO:0009119) | 2.68E-08 | 725 | 16 |
| **metabolism** | ribonucleoside triphosphate metabolic process (GO:0009199) | 3.28E-09 | 628 | 16 |
| **metabolism** | ribonucleotide metabolic process (GO:0009259) | 4.22E-08 | 748 | 16 |
| **metabolism** | ribose phosphate metabolic process (GO:0019693) | 4.47E-08 | 751 | 16 |
| **metabolism** | negative regulation of cellular protein metabolic process (GO:0032269) | 2.08E-09 | 509 | 15 |
| **metabolism** | negative regulation of protein metabolic process (GO:0051248) | 1.33E-08 | 582 | 15 |
| **metabolism** | GTP metabolic process (GO:0046039) | 7.05E-10 | 238 | 12 |
| **metabolism** | guanosine-containing compound metabolic process (GO:1901068) | 1.18E-09 | 249 | 12 |
| **regulation of catalysis** | regulation of catalytic activity (GO:0050790) | 1.16E-08 | 2008 | 25 |
| **regulation of catalysis** | regulation of protein modification process (GO:0031399) | 1.15E-10 | 1185 | 22 |
| **regulation of catalysis** | regulation of transferase activity (GO:0051338) | 1.38E-11 | 826 | 20 |
| **regulation of kinase activity** | regulation of kinase activity (GO:0043549) | 1.45E-11 | 719 | 19 |
| **regulation of kinase activity** | regulation of protein kinase activity (GO:0045859) | 8.71E-10 | 678 | 17 |
| **regulation of kinase activity** | positive regulation of kinase activity (GO:0033674) | 1.24E-07 | 469 | 13 |
| **regulation of kinase activity** | regulation of protein serine/threonine kinase activity (GO:0071900) | 2.04E-08 | 404 | 13 |
| **regulation of kinase activity** | regulation of phosphorylation (GO:0042325) | 3.59E-09 | 1122 | 20 |
| **regulation of kinase activity** | regulation of protein phosphorylation (GO:0001932) | 1.30E-09 | 931 | 19 |
| **regulation of kinase activity** | positive regulation of protein phosphorylation (GO:0001934) | 5.33E-07 | 642 | 14 |
| **regulation of mitochondrion organization** | regulation of mitochondrion organization (GO:0010821) | 2.15E-11 | 89 | 10 |
| **regulation of mitochondrion organization** | positive regulation of mitochondrion organization (GO:0010822) | 2.25E-11 | 58 | 9 |
| **regulation of mitochondrion organization** | regulation of mitochondrial outer membrane permeabilization involved in apoptotic signaling pathway (GO:1901028) | 7.51E-11 | 40 | 8 |
| **regulation of mitochondrion organization** | positive regulation of protein insertion into mitochondrial membrane involved in apoptotic signaling pathway (GO:1900740) | 3.01E-10 | 26 | 7 |
| **regulation of mitochondrion organization** | regulation of protein insertion into mitochondrial membrane involved in apoptotic signaling pathway (GO:1900739) | 3.01E-10 | 26 | 7 |
| **signalling** | response to stimulus (GO:0050896) | 2.07E-09 | ` | 46 |
| **signalling** | cellular response to stimulus (GO:0051716) | 5.98E-08 | 5446 | 39 |
| **signalling** | cell communication (GO:0007154) | 9.47E-09 | 4871 | 38 |
| **signalling** | signaling (GO:0023052) | 5.13E-09 | 4777 | 38 |
| **signalling** | single organism signaling (GO:0044700) | 5.13E-09 | 4777 | 38 |
| **signalling** | signal transduction (GO:0007165) | 2.65E-07 | 4356 | 34 |
| **signalling** | regulation of response to stimulus (GO:0048583) | 2.03E-10 | 2951 | 32 |
| **signalling** | response to chemical (GO:0042221) | 6.63E-08 | 3429 | 31 |
| **signalling** | response to stress (GO:0006950) | 7.64E-08 | 3004 | 29 |
| **signalling** | regulation of cell communication (GO:0010646) | 5.15E-08 | 2541 | 27 |
| **signalling** | regulation of signal transduction (GO:0009966) | 4.07E-09 | 2274 | 27 |
| **signalling** | regulation of signaling (GO:0023051) | 4.79E-08 | 2533 | 27 |
| **signalling** | intracellular signal transduction (GO:0035556) | 1.45E-11 | 1481 | 25 |
| **signalling** | Fc receptor signaling pathway (GO:0038093) | 1.32E-11 | 223 | 13 |
| **transport/localization** | localization (GO:0051179) | 1.03E-10 | 4227 | 38 |
| **transport/localization** | establishment of localization (GO:0051234) | 1.01E-11 | 3479 | 36 |
| **transport/localization** | transport (GO:0006810) | 5.08E-12 | 3404 | 36 |
| **transport/localization** | single-organism transport (GO:0044765) | 3.00E-12 | 2729 | 33 |
| **transport/localization** | cellular localization (GO:0051641) | 1.97E-16 | 1816 | 32 |
| **transport/localization** | establishment of localization in cell (GO:0051649) | 2.79E-15 | 1533 | 29 |
| **transport/localization** | intracellular transport (GO:0046907) | 2.95E-15 | 1140 | 26 |
| **transport/localization** | single-organism intracellular transport (GO:1902582) | 2.86E-16 | 924 | 25 |
| **transport/localization** | cellular component movement (GO:0006928) | 2.56E-10 | 1234 | 22 |
| **transport/localization** | vesicle-mediated transport (GO:0016192) | 1.86E-07 | 961 | 17 |
| **transport/localization** | cellular macromolecule localization (GO:0070727) | 8.92E-07 | 926 | 16 |
| **transport/localization** | cellular protein localization (GO:0034613) | 8.26E-07 | 921 | 16 |
| **transport/localization** | regulation of cellular localization (GO:0060341) | 2.65E-07 | 850 | 16 |
| **transport/localization** | regulation of intracellular transport (GO:0032386) | 2.22E-11 | 369 | 15 |
| **transport/localization** | regulation of establishment of protein localization (GO:0070201) | 3.61E-07 | 413 | 12 |
| **transport/localization** | cytoskeleton-dependent intracellular transport (GO:0030705) | 3.10E-08 | 86 | 8 |
