# Supplementary material for: Computational analysis of the LRRK2 interactome
Source: PeerJ. 2015 Feb 19;3:e778. doi: 10.7717/peerj.778 (PMC4338795; doi:10.7717/peerj.778)
Supplement: Table S5 — It contains specifications regarding the kinases in the LRRK2 interactome. Details are from the UniProt database (downloaded on the 9th January 2015). [file peerj-03-778-s005.docx]

| Kinases in the LRRK2 interactome | |
| --- | --- |
| MAP2K3 | Protein kinase superfamily. STE Ser/Thr protein kinase family. MAP kinase kinase subfamily. |
| MAP2K6 | Protein kinase superfamily. STE Ser/Thr protein kinase family. MAP kinase kinase subfamily. |
| MAP2K7 | Protein kinase superfamily. STE Ser/Thr protein kinase family. MAP kinase kinase subfamily. |
| GAK | Protein kinase superfamily. Ser/Thr protein kinase family. |
| PRKDC | PI3/PI4-kinase family. |
| LRRK1 | Protein kinase superfamily. TKL Ser/Thr protein kinase family. ROCO subfamily. |
| MATK | Protein kinase superfamily. Tyr protein kinase family. CSK subfamily. |
| NEK1 | Protein kinase superfamily. NEK Ser/Thr protein kinase family. NIMA subfamily. |
| PAK6 | Protein kinase superfamily. STE Ser/Thr protein kinase family. STE20 subfamily. |
| GSK3B | Protein kinase superfamily. CMGC Ser/Thr protein kinase family. GSK-3 subfamily. |
| AKT1 | Protein kinase superfamily. AGC Ser/Thr protein kinase family. RAC subfamily. |
